# Supplementary material for: Surveillance of cohesin-supported chromosome structure controls meiotic progression
Source: Nat Commun. 2020 Aug 28;11:4345. doi: 10.1038/s41467-020-18219-9 (PMC7455720; doi:10.1038/s41467-020-18219-9)
Supplement: Supplementary file 1 — Supplementary Information [file 41467_2020_18219_MOESM1_ESM.pdf]

**a**

| Genotype | Embryonic lethality (%) | n |
|---|---|---|
| WT (N2) | 2.32 | 1505 |
| *rec-8::GFP; rec-8 (ok978)* | 1.6 | 1664 |
| *rec-8$^{3XTEV}$::GFP; rec-8 (ok978)* | 2.44 | 1501 |
| *rec-8 (ok978)* | 86.89 | 929 |
| *coh-3::mcherry; coh-3 (gk112); coh-4 (tm1857)* | 2.41 | 1412 |
| *coh-3$^{3XTEV}$::mcherry; coh-3 (gk112); coh-4 (tm1857)* | 2.04 | 1306 |
| *coh-3 (gk112); coh-4 (tm1857)* | 97.57 | 774 |
| *rec-8$^{3XTEV}$; coh-3$^{3XTEV}$; coh-4 (tm1857)* | 2.28 | 1398 |
| *syp-2:AID; P$_{SUN-1}$::TIR-1::mRuby* | 2.48 | 1351 |
| *chk-2:AID; P$_{SUN-1}$::TIR-1::mRuby* | 4.71 | 871 |
| *chk-2 (me64)* | 98.17 | 821 |

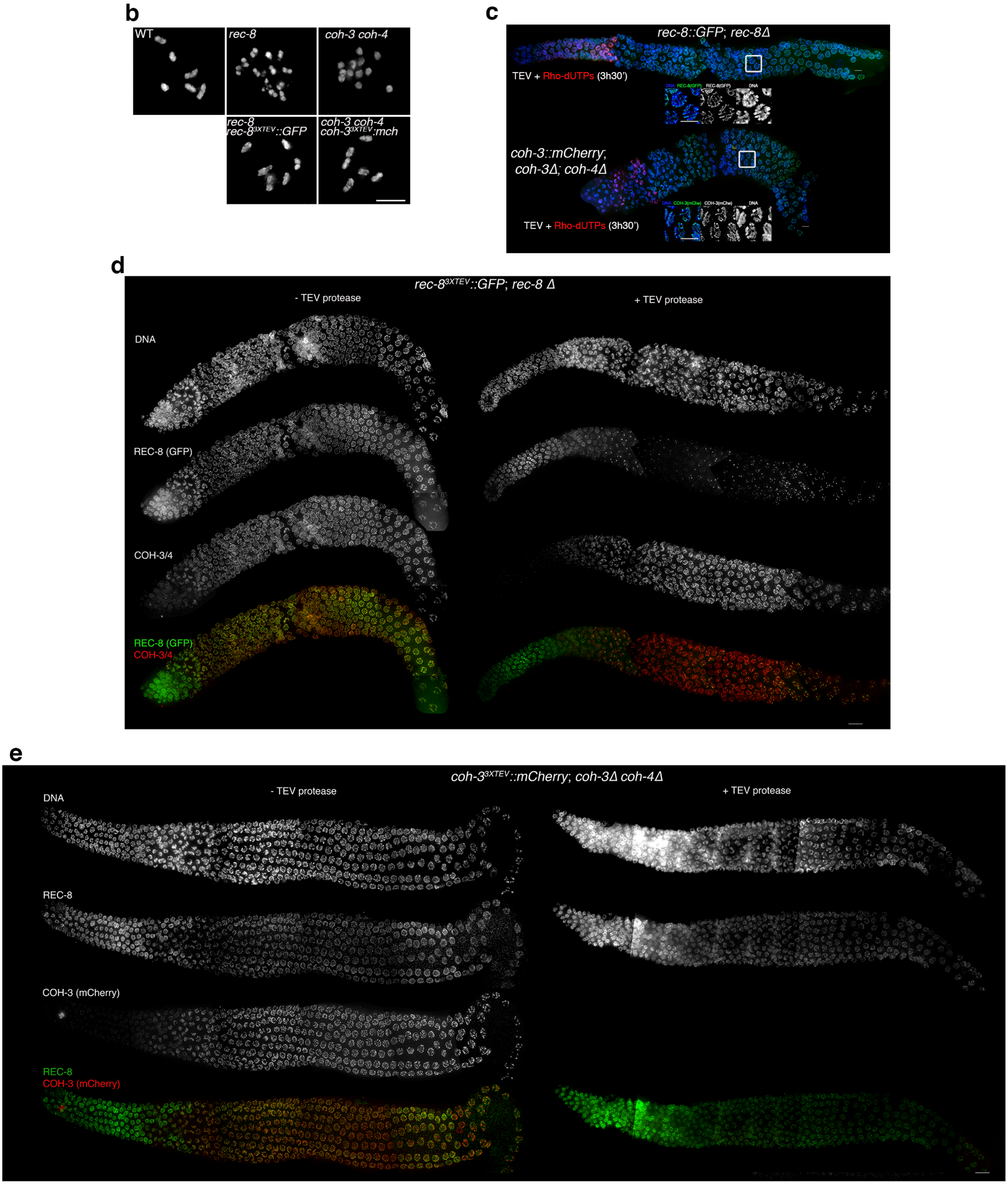

**Supplementary Fig. 1 (a)** Quantification of embryonic lethality in strains of indicated genotypes. Note that the *rec-8*$^{3XTEV}$*::GFP* and *coh-3*$^{3XTEV}$*::mCherry* transgenes rescue the high embryonic lethality of the *rec-8* single and *coh-3 coh-4* double mutants respectively. **(b)** Examples of diakinesis oocytes showing that expression of *rec-8*$^{3XTEV}$*::GFP* and *coh-3*$^{3XTEV}$*::mCherry* transgenes rescues chiasma formation in *rec-8* single and *coh-3 coh-4* double mutants respectively. **(c)** Injection of the TEV protease in germlines of worms expressing *rec-8::GFP* or *coh-3::mCherry* transgenes lacking the TEV recognition motif demonstrates normal cohesin localization and no apparent meiotic defects. The efficiency of the injection is confirmed by the incorporation of labelled nucleotides (Rho-dUTPs), which were co-injected with the TEV protease. Nuclei inside white rectangle are magnified on the inset shown below the germlines. **(d)** Whole germlines of indicated genotype before and after (3.5 hours) TEV protease injection demonstrating efficient removal of REC-8$^{3XTEV}$::GFP, while COH-3/4 remain associated with chromosomes. **(e)** Whole germlines of indicated genotype before and after (3.5 hours) TEV protease injection demonstrating efficient removal of COH-3$^{3XTEV}$::mCherry, while REC-8 remains associated with chromosomes. Data are representative of at least two independent experiments. Scale bar= 5 µm in all panels.

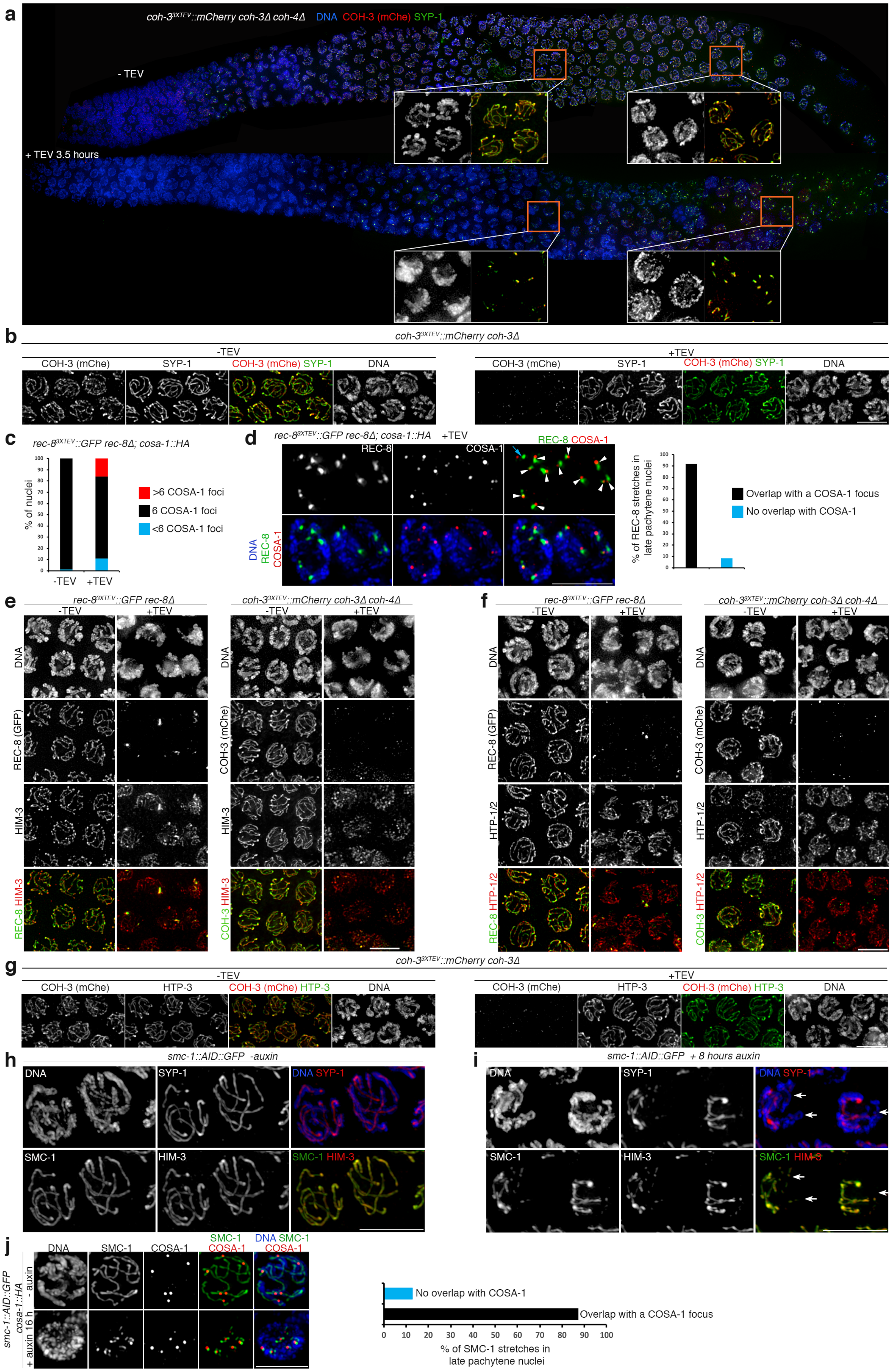

a
coh-3^3XTEV::mCherry coh-3Δ coh-4Δ DNA COH-3 (mChe) SYP-1
- TEV
+ TEV 3.5 hours
b
coh-3^3XTEV::mCherry coh-3Δ
-TEV
+TEV
COH-3 (mChe)
SYP-1
COH-3 (mChe) SYP-1
DNA
c
rec-8^3XTEV::GFP rec-8Δ; cosa-1::HA
% of nuclei
>6 COSA-1 foci
6 COSA-1 foci
<6 COSA-1 foci
-TEV
+TEV
d
rec-8^3XTEV::GFP rec-8Δ; cosa-1::HA +TEV
REC-8
COSA-1
REC-8 COSA-1
DNA REC-8 COSA-1
% of REC-8 stretches in late pachytene nuclei
Overlap with a COSA-1 focus
No overlap with COSA-1
e
rec-8^3XTEV::GFP rec-8Δ
coh-3^3XTEV::mCherry coh-3Δ coh-4Δ
-TEV
+TEV
DNA
REC-8 (GFP)
HIM-3
REC-8 HIM-3
COH-3 (mChe)
COH-3 HIM-3
f
rec-8^3XTEV::GFP rec-8Δ
coh-3^3XTEV::mCherry coh-3Δ coh-4Δ
HTP-1/2
REC-8 HTP-1/2
COH-3 HTP-1/2
g
coh-3^3XTEV::mCherry coh-3Δ
HTP-3
COH-3 (mChe) HTP-3
h
smc-1::AID::GFP -auxin
DNA
SYP-1
DNA SYP-1
SMC-1
HIM-3
SMC-1 HIM-3
i
smc-1::AID::GFP + 8 hours auxin
j
smc-1::AID::GFP cosa-1::HA
- auxin
+ auxin 16 h
DNA
SMC-1
COSA-1
SMC-1 COSA-1
DNA SMC-1 COSA-1
No overlap with COSA-1
Overlap with a COSA-1 focus
% of SMC-1 stretches in late pachytene nuclei

**Supplementary Fig. 2 (a)** Whole germlines of indicated genotype before and after (3.5 hours) TEV protease injection showing that COH-3$^{3XTEV}$::mCherry removal induces SC disassembly. Note that some small SYP-1 signals persist colocalising with COH-3$^{3XTEV}$::mCherry signals that are more prominent in late pachytene nuclei, where their number is close to 6. Nuclei inside white rectangle are magnified on the inset shown below the germlines. **(b)** TEV-mediated removal of COH-3$^{3XTEV}$::mCherry from pachytene nuclei of worms expressing endogenous COH-4 does not induce SC disassembly. Images on the +TEV panel were acquired 3.5 hours after TEV injection. Note efficient removal of COH-3$^{3XTEV}$::mCherry and persistence of SYP-1 tracks. **(c)** Quantification of COSA-1 foci in late pachytene nuclei of *rec-8*$^{3XTEV}$*::GFP rec-8Δ; cosa-1::HA* 3.5 hours after TEV injection (99 nuclei were analysed). **(d)** Late pachytene nuclei of *rec-8*$^{3XTEV}$*::GFP rec-8Δ; cosa-1::HA* 3.5 hours after TEV injection showing six REC-8 stretches. Note that 11 out 12 REC-8 stretches overlap with a COSA-1 focus (white arrowheads), the only REC-8 stretch that does not overlap with a COSA-1 focus (blue arrow) is in close proximity to COSA-1 focus. Graph shows quantification of REC-8 stretches overlapping with a COSA-1 focus 3.5 hours after TEV injection (82 nuclei containing a total of 467 REC-8 stretches). **(e-f)** HORMA proteins HIM-3 (e) and HTP-1/2 (f) remain associated with axial elements following TEV-mediated removal of REC-8$^{3XTEV}$::GFP or COH-3$^{3XTEV}$::mCherry. **(g)** HTP-3 remains associated with axial elements following TEV-mediated removal of COH-3$^{3XTEV}$::mCherry from pachytene nuclei of worms expressing endogenous COH-4. Images on the +TEV panel were acquired 3.5 hours after TEV injection. **(h-i)** Auxin-mediated depletion of SMC-1::AID::GFP for 8 hours induces partial loss of cohesin from chromosomes. Note that regions lacking SMC-1 staining also lack HIM-3 and SYP-1 staining (arrowheads in i). Images were acquired using a structural illumination microscope. **(j)** Late pachytene nuclei of *smc-1::AID::GFP; cosa-1::HA* before and after 16 hours of auxin treatment. Note that following auxin treatment short stretches of SMC-1 overlap with COSA-1 foci. Graph shows quantification of SMC-1 stretches overlapping with a COSA-1 focus after 16 hours of auxin treatment (37 nuclei containing a total of 236 SMC-1 stretches). Data are representative of at least two independent experiments. Scale bar= 5 µm in all panels. Source data for graphs in panels c, d, and j are provided as Source Data file.

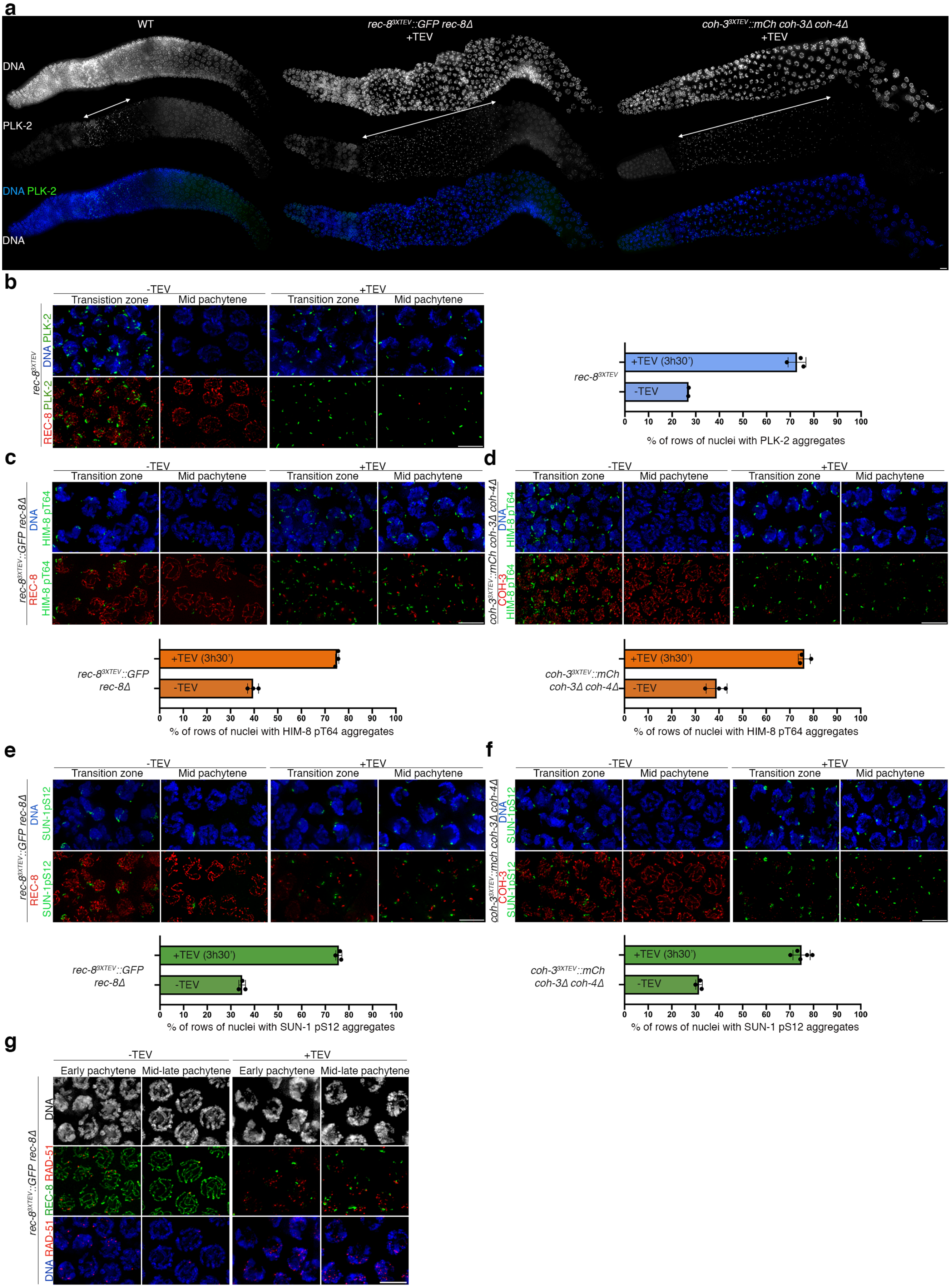


**Supplementary Fig. 3 (a)** Whole germlines of indicated genotypes showing PLK-2 localization. Double-headed arrows indicates the beginning and end of regions containing nuclei with PLK-2

aggregates on the nuclear envelope. Note that in WT germlines PLK-2 aggregates are only present in the transition zone, while germlines stained 3.5 hours after TEV-mediated removal of REC-8$^{3XTEV}$::GFP or COH-3$^{3XTEV}$::mCherry contain nuclei with PLK-2 aggregates through most of the pachytene region. **(b)** TEV-mediated removal of REC-8$^{3XTEV}$ (CRISPR strain with 3XTEV added to endogenous *rec-8* locus) induces reappearance of PLK-2 aggregates on the nuclear envelope of pachytene nuclei 3.5 hours after TEV injection. **(c-f)** TEV-mediated removal of REC-8$^{3XTEV}$::GFP or COH-3$^{3XTEV}$::mCherry induces reappearance of HIM-8 pT64 (c-d) and SUN-1 pS12 (e-f) signals on the nuclear envelope of pachytene nuclei. Graphs show percentage of vertical rows of nuclei between meiotic onset and end of pachytene in which over 50% of nuclei contained more than 1 aggregate of SUN-1 pS12 or HIM-8 pT64 on the nuclear envelope. **(g)** TEV-mediated removal of REC-8$^{3XTEV}$::GFP induces accumulation of RAD-51 foci in pachytene nuclei (example of one of the three germlines included in graph shown in Fig. 3e). Three germlines were quantified per condition in graphs shown in b-f (except for the -TEV control in panel B where two germlines were used and the + TEV of panel f where five germlines were used), data are presented as mean values ± standard deviations. Scale bar= 5 µm in all panels. Source data for graphs in panels b, c, d, e, and f are provided as Source Data file.

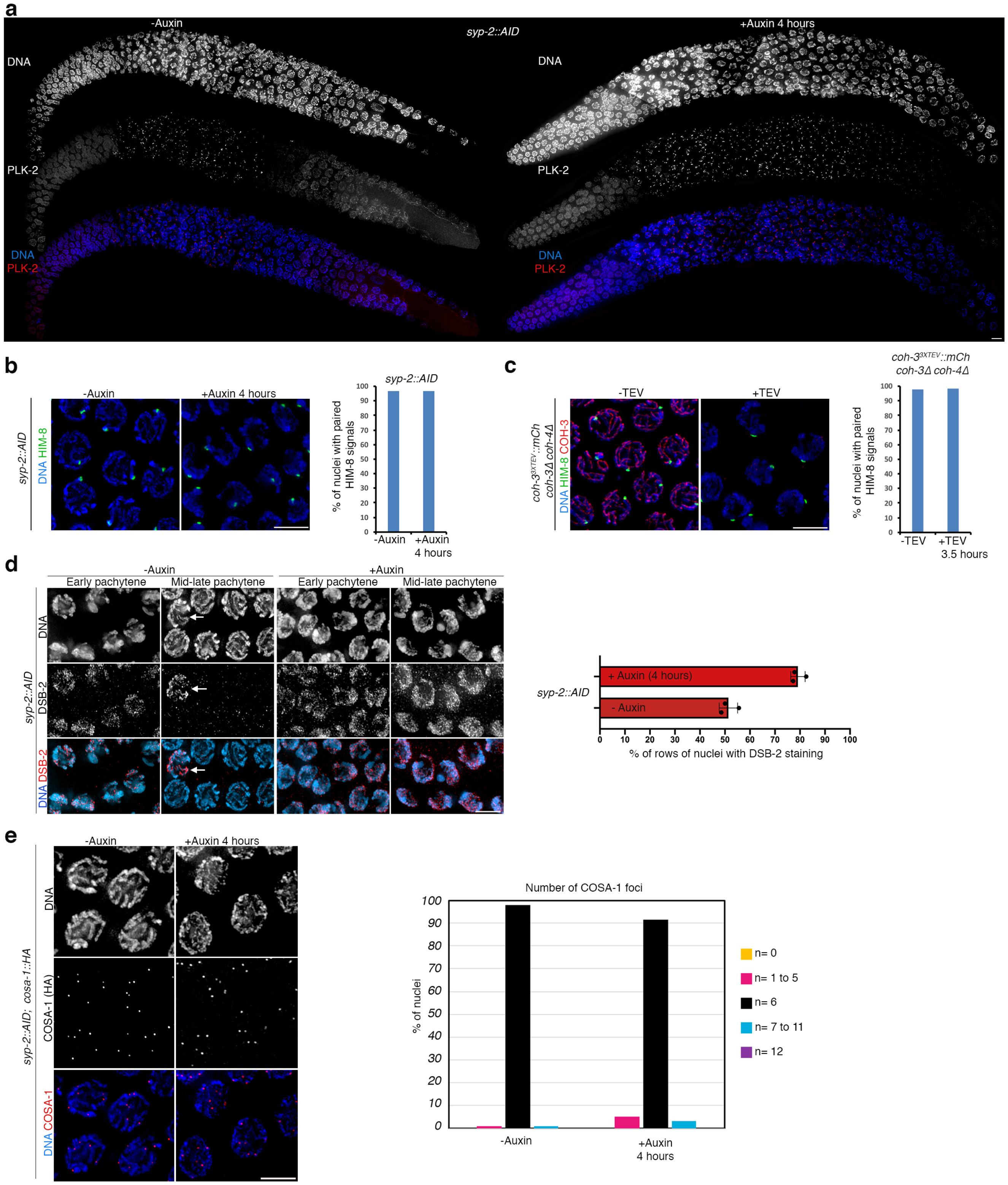


**Supplementary Fig. 4 (a)** Whole germlines of *syp-2::AID* worms before and after (4 hours) auxin treatment, which induces appearance of PLK-2 aggregates on the nuclear envelope in most pachytene nuclei (examples of one of the three germlines per genotype that were scored in the graph of Fig. 4b). **(b-c)** X chromosomes pairing centers (visualised using anti-HIM-8 antibodies) are associated following auxin-mediated SYP-2 depletion (b) or COH-3 TEV-mediated cleavage (c). **(d)** Auxin-mediated depletion of SYP-2 causes de novo association of DSB-2 with pachytene

chromosomes. Quantification indicates percentage of vertical rows of nuclei between meiotic onset and the end of pachytene in which over 50% of nuclei were positive for DSB-2 (three germlines were scored per genotype, data are presented as mean values ± standard deviations). **(e)** Late pachytene nuclei from *syp-2::AID; cosa-1::HA* worms showing that COSA-1 foci persist after 4 hours of auxin treatment. Graph shows quantification of COSA-1::HA foci in late pachytene nuclei from 5 germlines of *syp-2::AID; cosa-1::HA* worms before and after auxin treatment. Note that most nuclei show 6 foci before and after auxin treatment. Nuclei from at least three germlines were included in all the quantifications. Scale bar= 5 µm in all panels. Source data for graphs in panels b, c, d, and e are provided as Source Data file.

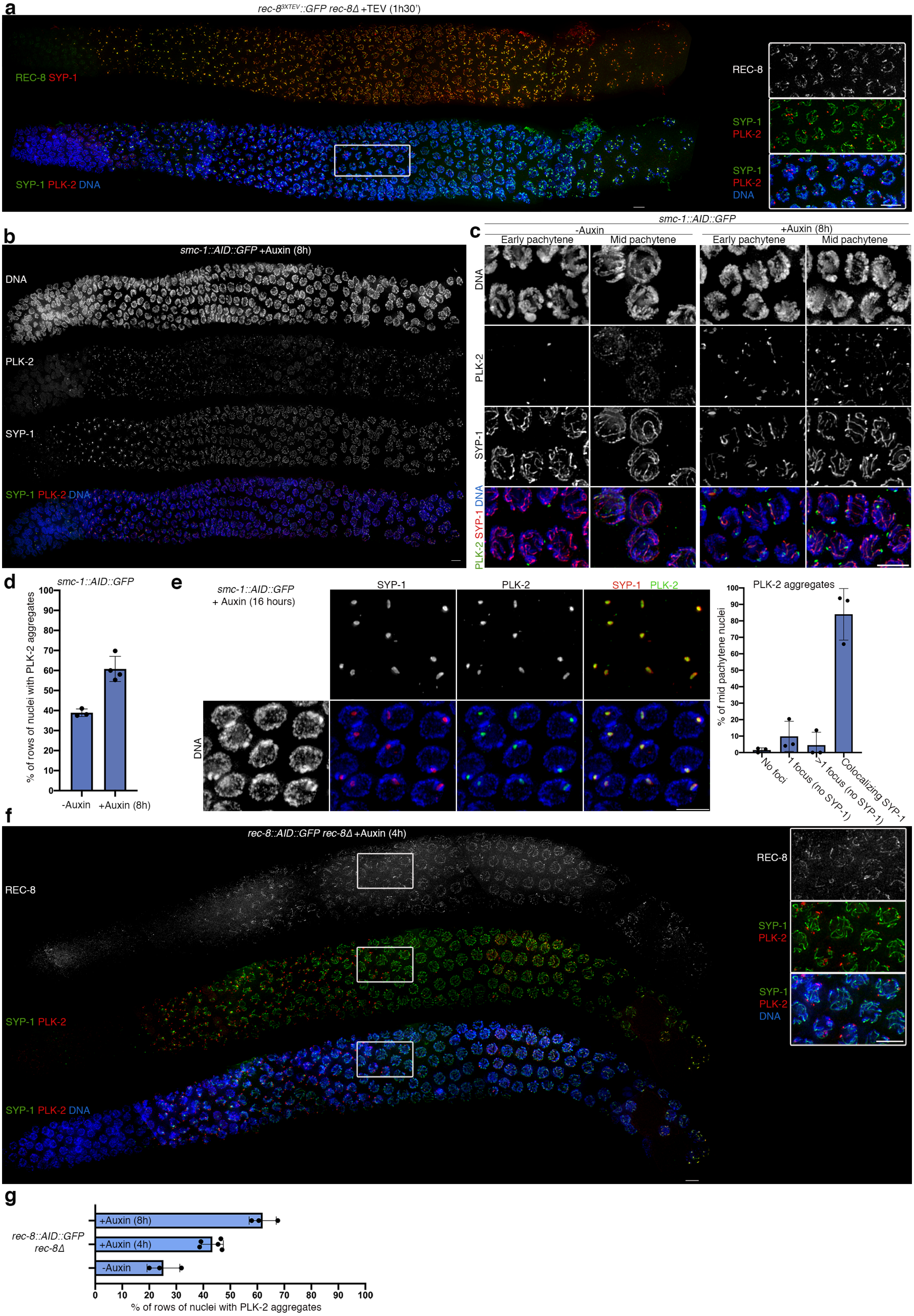

a
rec-8^3XTEV::GFP rec-8Δ +TEV (1h30')
REC-8 SYP-1
SYP-1 PLK-2 DNA
REC-8
SYP-1 PLK-2
SYP-1 PLK-2 DNA
b
smc-1::AID::GFP +Auxin (8h)
DNA
PLK-2
SYP-1
SYP-1 PLK-2 DNA
c
smc-1::AID::GFP
-Auxin
+Auxin (8h)
Early pachytene
Mid pachytene
Early pachytene
Mid pachytene
DNA
PLK-2
SYP-1
PLK-2 SYP-1 DNA
d
smc-1::AID::GFP
% of rows of nuclei with PLK-2 aggregates
-Auxin
+Auxin (8h)
e
smc-1::AID::GFP
+ Auxin (16 hours)
SYP-1
PLK-2
SYP-1 PLK-2
DNA
PLK-2 aggregates
% of mid pachytene nuclei
No foci
1 focus (no SYP-1)
>1 focus (no SYP-1)
Colocalizing SYP-1
f
rec-8::AID::GFP rec-8Δ +Auxin (4h)
REC-8
SYP-1 PLK-2
SYP-1 PLK-2 DNA
REC-8
SYP-1 PLK-2
SYP-1 PLK-2 DNA
g
rec-8::AID::GFP rec-8Δ
+Auxin (8h)
+Auxin (4h)
-Auxin
% of rows of nuclei with PLK-2 aggregates

**Supplementary Fig. 5 (a)** Whole germline from *rec-8*$^{3XTEV}$*::GFP rec-8Δ* dissected 1.5 hours post TEV injection. Note partial REC-8 removal and SC disassembly (SYP-1) in mid pachytene nuclei that display multiple PLK-2 aggregates on the nuclear envelope. Nuclei inside white rectangle are magnified on the inset shown on the right-hand side of the panel. Data are representative of two independent experiments. **(b-d)** 8 hours of auxin-mediated depletion of SMC-1::AID::GFP causes partial SC disassembly and appearance of PLK-2 aggregates in mid pachytene nuclei. Note that SC disassembly is more prominent in transition zone and early pachytene nuclei. Graph shows percentage of vertical rows of nuclei between meiotic onset and end of pachytene in which over 50% of nuclei contained more than 1 PLK-2 aggregate. Three germlines were scored per genotype, data are presented as mean values ± standard deviations. **(e)** 16 hours of auxin treatment in *smc-1::AID::GFP* worms causes full SC disassembly and localization of PLK-2 to SYP-1 aggregates. Graph shows the percentage of mid pachytene nuclei with indicated patterns of PLK-2 staining. Note that in 84% of nuclei PLK-2 is only found colocalizing with SYP-1 aggregates and that only 4% of nuclei display more than 1 PLK-2 aggregate localizing to the nuclear envelope. A total of 131 mid pachytene nuclei from 3 germlines are included in the graph (data are presented as mean values ± standard deviations). **(f)** Whole germline from *rec-8::AID::GFP rec-8Δ* dissected after 4 hours of auxin treatment. Note partial REC-8::AID::GFP depletion and SC disassembly in mid pachytene nuclei that display PLK-2 aggregates on the nuclear envelope. Nuclei inside white rectangle are magnified on the inset shown on the right-hand side of the panel (example of one of the three germlines quantified in (g). **(g)** Graph shows percentage of vertical rows of nuclei between meiotic onset and end of pachytene in which over 50% of nuclei contained more than 1 PLK-2 aggregate after 0, 4, and 8 hours of auxin treatment. Three germlines were quantified per condition (except four germlines for + auxin in panel d and five germlines for + auxin (4 hours) in panel g), data are presented as mean values ± standard deviations. Scale bar= 5 µm in all panels. Source data for graphs in panels d, e, and g are provided as Source Data file.

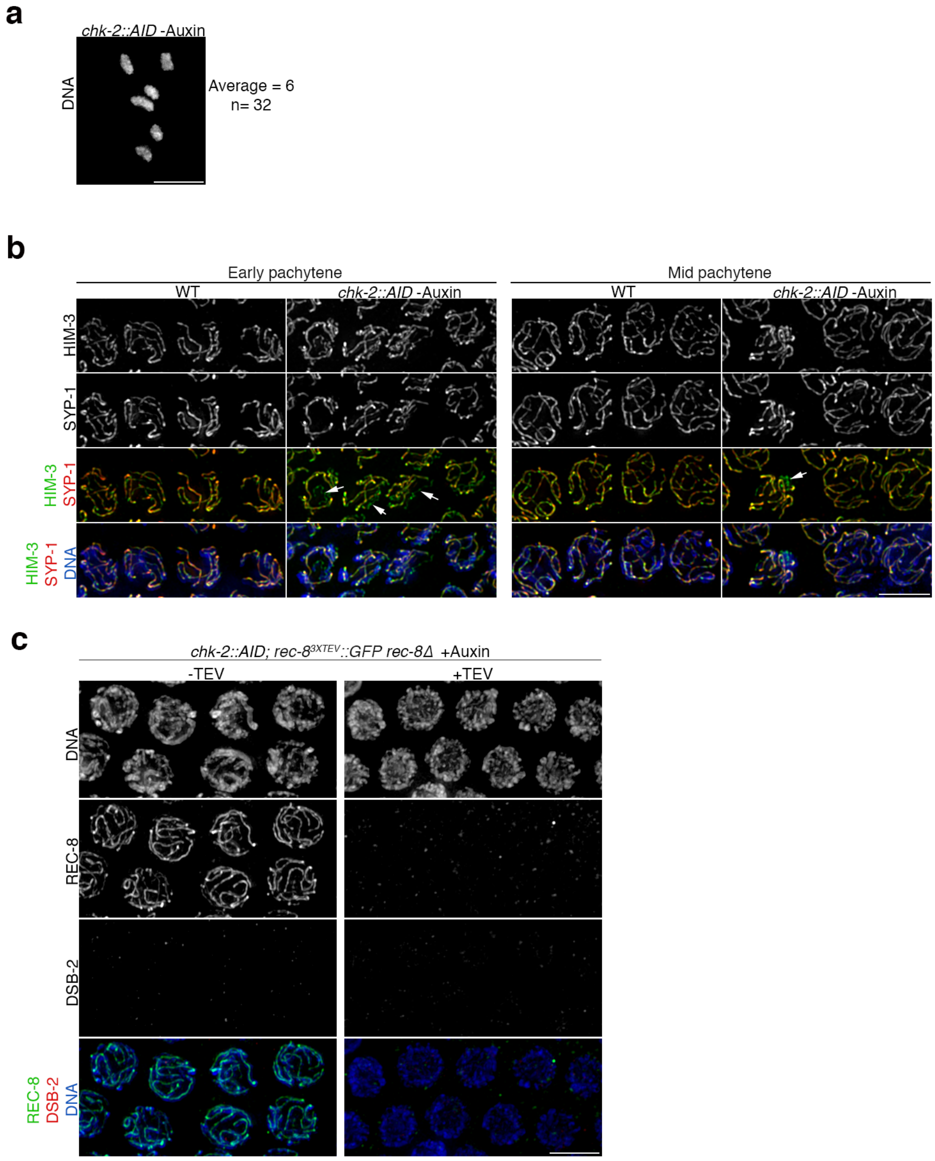


**Supplementary Fig. 6 (a)** Worms homozygous for *chk-2::AID* and carrying the TIR1 transgene display normal chiasma formation in the absence of auxin, evidenced by the presence of six DAPI-stained bodies in diakinesis nuclei. **(b)** Homozygous *chk-2::AID* worms carrying the TIR1 transgene display delayed SC assembly in the absence of auxin. Arrowheads point to unsynapsed axial elements displaying HIM-3 signals but no SYP-1 staining. **(c)** TEV-mediated removal of REC-8 from pachytene nuclei following auxin-dependent CHK-2 depletion during 6 hours fails to induce reappearance of DSB-2. Experimental design was the same as that described in Fig. 5c: CHK-2 was depleted by exposing worms to 6 hours of auxin treatment before TEV injection and germlines were dissected and fixed 3 hours post TEV injection. Data are representative of two independent experiments. Scale bar= 5 µm in all panels.

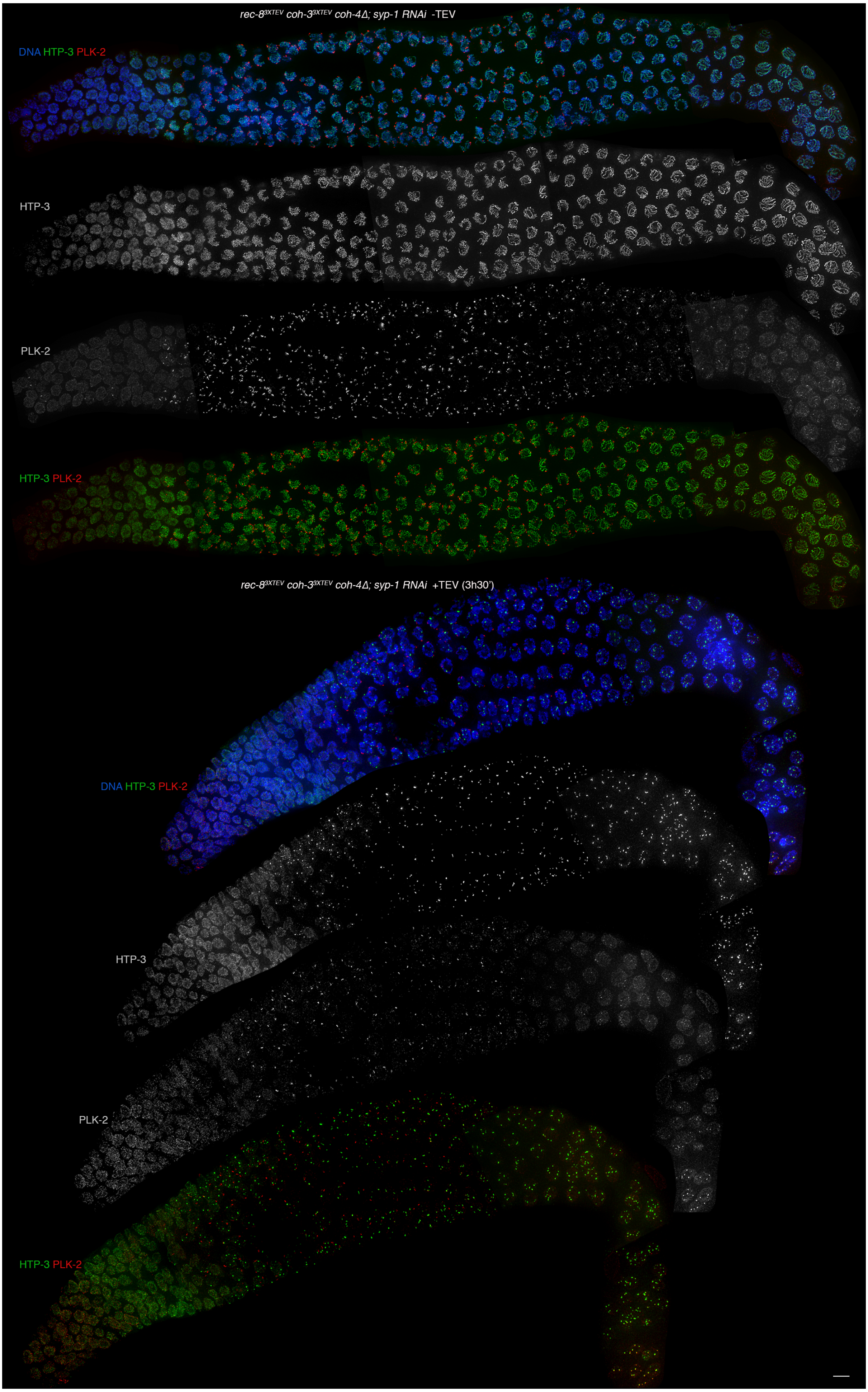
rec-8$^{3XTEV}$ coh-3$^{3XTEV}$ coh-4Δ; syp-1 RNAi -TEV
DNA HTP-3 PLK-2
HTP-3
PLK-2
HTP-3 PLK-2
rec-8$^{3XTEV}$ coh-3$^{3XTEV}$ coh-4Δ; syp-1 RNAi +TEV (3h30')
DNA HTP-3 PLK-2
HTP-3
PLK-2
HTP-3 PLK-2

**Supplementary Fig. 7** Germlines from *rec-8$^{3XTEV}$ coh-3$^{3XTEV}$ coh-4Δ; syp-1RNAi* worms before (top) and after (bottom) TEV injection. Note intact HTP-3 axial elements and the presence of multiple PLK-2 aggregates on the nuclear envelope before TEV injection. TEV injection induces large disassembly of axial elements, visualised by the accumulation of HTP-3 in small nuclear aggregates, and a reduction both in the number of nuclei displaying PLK-2 aggregates as well as in the number of PLK-2 aggregates per nucleus. Examples of one of the three germlines per condition (-TEV and +TEV included on the graph shown in Fig. 7c).

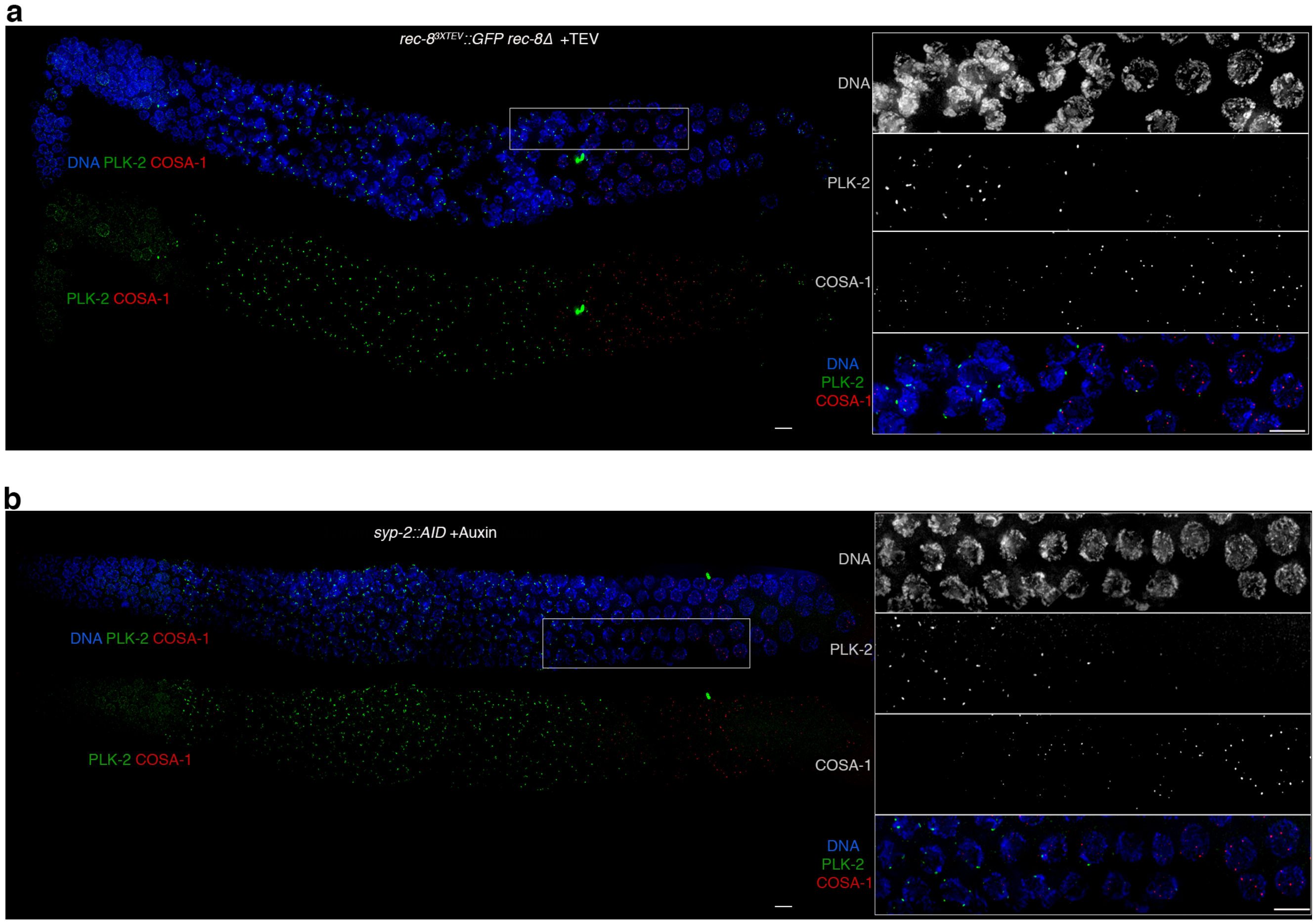


**Supplementary Fig. 8 (a)** Whole germline from *rec-8*$^{3XTEV}$*::GFP rec-8Δ; cosa-1::HA* worms dissected 3.5 hours post TEV injection. Note that PLK-2 aggregates are lacking in late pachytene nuclei displaying bright COSA-1 foci. Nuclei inside white rectangle are magnified on the inset shown on the right-hand side of the panel. **(b)** Whole germline from *syp-2::AID; cosa-1::HA* worms dissected after 4 hours of auxin treatment. Note that PLK-2 aggregates are lacking in late pachytene nuclei displaying bright COSA-1 foci. Nuclei inside white rectangle are magnified on the inset shown on the right-hand side of the panel. Data are representative of two independent experiments. Scale bar= 5 µm in all panels.

| Strain | Genotype |
|---|---|
| ATGSi355 | *fqSi16 II ; rec-8(ok978) IV* |
| ATGSi441 | *fqSi15 II ; coh-3(gk112) coh-4(1857) V* |
| ATG415 | *smc-1 (fq64[smc-1::AID::GFP]) I ; ieSi38 IV* |
| ATGSi392 | *fqSi16 II ; rec-8(ok978) spo-11(ok79) IV / nT1 [qls51] (IV;V)* |
| ATG298 | *syp-2 (fq30[syp-2::AID]) V ; ieSi38 IV* |
| ATG506 | *syp-3 (syb1022[mScarlet::syp-3]) I ; fqSi13 II ; syp-2 (fq30[syp-2::AID]) V ; ieSi38 IV* |
| ATG330 | *chk-2 (fq41[chk-2::AID]) V ; ieSi38 IV* |
| ATG387 | *chk-2 (fq41[chk-2::AID]) V ; fqSi16 II ; rec-8(ok978) ieSi38 IV* |
| ATGSi525 | *fqSi15 II ; coh-3(gk112) coh-4(1857) V ; htp-1(gk174) IV / nT1 [unc-? (n754) let-? qls50] (IV;V)* |
| ATG302 | *rec-8(fq32[rec-8*$^{3XTEV}$*]) IV ; coh-3(fq35[coh-3*$^{3XTEV}$*]) coh-4 (1857) V* |
| ATGSi578 | *fqSi16 II ; rec-8(ok978) IV ; cosa-1(fq42[cosa-1::HA]) III* |
| ATG169a | *fqSi18 I ; coh-3(gk112) coh-4(1857) V* |
| ATG554 | *syp-2 (fq30[syp-2::AID]) V ; cosa-1(fq42[cosa-1::HA]) III ; ieSi38 IV* |
| ATG323 | *fqSi17 II ; rec-8(ok978) IV ; ieSi38IV* |
| ATGSi468 | *fqSi15 II ; coh-3(gk112)V* |
| ATG564 | *smc-1 (fq64[smc-1::AID::GFP]) I ; cosa-1 (fq42[cosa-1::HA]) III; ieSi38 IV* |
| ATG614 | *fqSi13 II; syp-2(ok307) V/ nT1 [unc-? (n754) let-? qls50] (IV;V)* |
| ATG325 | *rec-8(fq32[rec-8*$^{3XTEV}$*]) IV* |

**Supplementary Table 1. List of *C. elegans* strains created in this study.**

| Transgene | Genotype | Origin |
|---|---|---|
| *fqSi16* | [*Prec-8 rec-8$^{3XTEV}$::GFP 3'UTR rec*-8; *cb-unc-119(+)*] | This study |
| *fqSi15* | [*Pcoh-3 coh-3*::$^{3XTEV}$*::mCherry 3'UTR coh-3*; *cb-unc-119(+)*] | This study |
| *fqSi13* | [*Pplk-2 plk-2*::GFP *3'UTR plk-2*; *cb-unc-119(+)*] | This study |
| *fqSi23* | [*Prec-8 rec-8::GFP 3'UTR rec*-8; *cb-unc-119(+)*] | Crawley *et al.,* 2016 [1] |
| *fqSi18* | [*Pcoh-3 coh-3*::*mCherry 3'UTR coh-3*; *cb-unc-119(+)*] | This study |
| *fqSi17* | [*Prec-8 rec-8::AID::GFP 3'UTR rec*-8; *cb-unc-119(+)*] | This study |
| *ieSi38* | [*Psun-1 TIR1::mRuby 3'UTR sun-1; cb-unc-119(+)*] | Zhang *et al.*, 2015 [2] |

**Supplementary Table 2. Transgenes used in this study.**

| Alelle | sgRNA | Repair template |
|---|---|---|
| *rec-8(fq32[rec-8<sup>3XTEV</sup>])* | CTGGGGTGCTGGTTGTTCCA | ttgtctctattgctctgctcccgagtgaaaccgtggagcaggagaatttgtatt<br>ttcagggtgcttctgaaaacctttacttccaaggagagctcgaa<br>aatctttatttccagggaccagcaccccaggagcctattcaagagcccattcaa |
| *Coh-3(fq35[coh-3<sup>3XTEV</sup>])* | GGAACTTTGGGAATTCTTAT | ctcggaaaacatggatctagatgacgaagtttcgattgagaatttgtatt<br>ttcagggtgcttctgaaaacctttacttccaaggagagctcg<br>aaaatctttatttccagggaccgatcaggattcccaaagttcctcttagt<br>gctacaaagaaacaaa |
| *syp-2(fq30[syp-2::AID])* | ATTTATAACTTGTCAGCCCA | Left oligo:<br>aacttctggatttggttgaaacgctcgagccgtgggcagataaattg<br>atgcctaaagatccagccaaacctccggccaaggcacaagttgtgggatggccaccggtga<br>gatcataccggaagaac<br>Right oligo:<br>tgggatggccaccggtgagatcataccggaagaacgtgatggtttcctgccaaaaatcaagcg<br>gtggcccggaggcggcggcgttcgtgaagtaaatcatctgttgtattcaatttccttgttttattacat |
| *chk-2(fq41[chk-2::AID])* | AATGTGAACAACGTTCCACG | Left oligo:<br>aaaatttcaatttttttagccttaaaaaccctattttcaggcaaaaatgggaggtggagg<br>tggagctatgcctaaagatccagccaaacctccggccaaggcacaagttgtgggatggcca<br>ccggtgagatcataccggaagaac<br>Right oligo:<br>tgggatggccaccggtgagatcataccggaagaacgtgatggtttcctgccaaaaatcaagc<br>ggtggcccggaggcggcggcgttcgtgaagtagacaacgttccacgtgggtccccaccactt<br>caccgccgcaaaagtccg |
| *cosa-1(fq42[cosa-1::HA])* | TGTCAGAGATGGTAGTTACG | cagaatgagagtattccggaatgcagcacctcctcgtacccatacgatgtt<br>ccagattacgcttaactaccatctctgacagcacctctttgtcgccgat |
| *smc-1(fq64[smc-1::AID::GFP])* | TCCATAGCTACAATGAGTAA | Left oligo:<br>Tgactgaaaatcctcctactccttccatagctacaatgccaaaagatccagcc<br>Aaacctccggccaaggcacaagttgtgggatggccaccggtgagatcataccggaagaac<br><br>Right oligo:<br>tgggatggccaccggtgagatcataccggaagaacgtgatggtttcctgccaaaaatcaag<br>cggtggcccggaggcggcggcgttcgtgaagatgTCtaaaggagaagaacttttcactggagttgtcccaa |

**Supplementary Table 3. sgRNAs and repair templates used for the CRISPR alleles created in this study.**

| Genotype | Zone 1 | Zone 2 | Zone 3 | Zone 4 | Zone 5 | Zone 6 |
|---|---|---|---|---|---|---|
| *rec-8$^{3XTEV}$::GFP; rec-8* (-TEV) | 131 | 135 | 163 | 160 | 133 | 120 |
| *rec-8$^{3XTEV}$::GFP; rec-8* (+TEV) | 172 | 166 | 185 | 146 | 126 | 113 |
| *rec-8$^{3XTEV}$::GFP; rec-8; spo-11* (-TEV) | 114 | 124 | 154 | 144 | 133 | 115 |
| *rec-8$^{3XTEV}$::GFP; rec-8; spo-11* (+TEV) | 117 | 143 | 136 | 137 | 124 | 114 |
| *syp-2::AID* (-Auxin) | 241 | 165 | 215 | 169 | 139 | 172 |
| *syp-2::AID* (+Auxin) | 174 | 194 | 172 | 166 | 155 | 98 |

**Supplementary Table 4. Number of nuclei analysed for RAD-51 staining in each region of the gonad in different genotypes**

## References


1. Crawley, O. *et al.* Cohesin-interacting protein WAPL-1 regulates meiotic chromosome structure and cohesion by antagonizing specific cohesin complexes. *Elife* **5**, e10851 (2016).
2. Zhang, L., Ward, J.D., Cheng, Z. & Dernburg, A.F. The auxin-inducible degradation (AID) system enables versatile conditional protein depletion in C. elegans. *Development* **142**, 4374-4384 (2015).
