# Supplementary material for: Surveillance of cohesin-supported chromosome structure controls meiotic progression
Source: Nat Commun. 2020 Aug 28;11:4345. doi: 10.1038/s41467-020-18219-9 (PMC7455720; doi:10.1038/s41467-020-18219-9)
Supplement: Supplementary file 3 — Description of Additional Supplementary Files [file 41467_2020_18219_MOESM3_ESM.pdf]

## Description of Additional Supplementary Files

File Name: Supplementary Movie 1
Description: *In vivo* imaging of the transition zone and early pachytene regions of the germline from a *mScarlet::syp-3; syp-2::AID; fqSi13[PLK-2::GFP]* worm (not treated with auxin). Note the presence of dynamic PLK-2::GFP aggregates (green) on the nuclear envelope of transition zone nuclei at the bottom left corner of the video and the presence of SC tracks (red) on all pachytene nuclei. Filming corresponds to 7 minutes.

File Name: Supplementary Movie 2
Description: *In vivo* imaging of the mid and late pachytene regions (left to right on the video) of the germline from a *mScarlet::syp-3; syp-2::AID; fqSi13[PLK-2::GFP]* worm (not treated with auxin). Note absence of PLK-2::GFP aggregates (green) and the presence of SC tracks (red) on all nuclei. Filming corresponds to 7 minutes.

File Name: Supplementary Movie 3
Description: *In vivo* imaging of the mid and late pachytene regions (left to right on the video) of the germline from a *mScarlet::syp-3; syp-2::AID; fqSi13[PLK-2::GFP]* worm after 2 hours of auxin treatment. Note presence of dynamic PLK-2::GFP aggregates (green) on the nuclear envelop of most nuclei, apart from late pachytene nuclei at the bottom right-hand corner, and the absence of SC tracks (red) on all nuclei. Filming corresponds to 7 minutes.

File Name: Supplementary Movie 4
Description: Zoomed pachytene nucleus from video S3 (*mScarlet::syp-3; syp-2::AID; fqSi13[PLK-2::GFP]* after 2 hours of auxin treatment). Note that PLK-2::GFP aggregates (green) undergo fusion and splitting events.

File Name: Supplementary Movie 5
Description: Tracking of PLK-2::GFP aggregates over 7 minutes at 5 seconds intervals in a pachytene nucleus of *syp-2::AID; fqSi13[PLK-2::GFP]* following 2 hours of auxin treatment. Tracks are colour coded to indicate average speed of the aggregate during the indicated trajectory according to the scale included at the bottom of the movie. A final projection of this movie is shown in Fig. 4d.

File Name: Supplementary Movie 6
Description: Tracking of PLK-2::GFP aggregates over 7 minutes at 5 seconds intervals in a pachytene nucleus of *syp-2(ok307)* mutant carrying the *fqSi13[PLK-2::GFP]* transgene. Tracks are colour coded to indicate average speed of the aggregate during the indicated trajectory according to the scale included at the bottom of the movie. A final projection of this movie is shown in Fig. 4d.

File Name: Supplementary Data 1
Description: **Changes in gene expression of 335 meiotic genes following auxin-mediated depletion of SC component SYP-2 compared to untreated controls.**

baseMean: average of the normalized count values for all samples (is a just the average of the normalized count values, dividing by size factors, taken over all samples).

log2FoldChange: effect size estimate (Treatment vs Control = how much the gene expression has changed due to Treatment). It is reported as log scale to base 2 (a value of 1.5 means 2^1.5 = 2.82 increase in expression)

lfcSE: uncertainty associated with log2FoldChange

stat: Wald statistic

pvalue: Two tailed Wald test p-value. probability that observed log2FoldChange would be observed under null hypothesis

padj: BH adjusted p-values. Fraction of false positives given a gene’s pvalue (method = Benjamini-Hochberg)
