# Supplementary material for: Surveillance of cohesin-supported chromosome structure controls meiotic progression
Source: Nat Commun. 2020 Aug 28;11:4345. doi: 10.1038/s41467-020-18219-9 (PMC7455720; doi:10.1038/s41467-020-18219-9)
Supplement: Supplementary file 11 — Reporting Summary [file 41467_2020_18219_MOESM11_ESM.pdf]

nature research

Corresponding author(s): Enrique Martinez-Perez

Last updated by author(s): Jul 2, 2020

Reporting Summary

Nature Research wishes to improve the reproducibility of the work that we publish. This form provides structure for consistency and transparency in reporting. For further information on Nature Research policies, see our Editorial Policies and the Editorial Policy Checklist.

Statistics

For all statistical analyses, confirm that the following items are present in the figure legend, table legend, main text, or Methods section.

n/a	Confirmed	
☐	☒	The exact sample size (n) for each experimental group/condition, given as a discrete number and unit of measurement
☐	☒	A statement on whether measurements were taken from distinct samples or whether the same sample was measured repeatedly
☐	☒	The statistical test(s) used AND whether they are one- or two-sided<br>*Only common tests should be described solely by name; describe more complex techniques in the Methods section.*
☒	☐	A description of all covariates tested
☒	☐	A description of any assumptions or corrections, such as tests of normality and adjustment for multiple comparisons
☐	☒	A full description of the statistical parameters including central tendency (e.g. means) or other basic estimates (e.g. regression coefficient) AND variation (e.g. standard deviation) or associated estimates of uncertainty (e.g. confidence intervals)
☐	☒	For null hypothesis testing, the test statistic (e.g. F, t, r) with confidence intervals, effect sizes, degrees of freedom and P value noted<br>*Give P values as exact values whenever suitable.*
☒	☐	For Bayesian analysis, information on the choice of priors and Markov chain Monte Carlo settings
☒	☐	For hierarchical and complex designs, identification of the appropriate level for tests and full reporting of outcomes
☒	☐	Estimates of effect sizes (e.g. Cohen's d, Pearson's r), indicating how they were calculated

Our web collection on statistics for biologists contains articles on many of the points above.

Software and code

Policy information about availability of computer code

Data collection	SoftWoRx 3.0 (Applied Precision); ZEN Black 2012 SP4, version 13.0.2.518 (Carl Zeiss Microimaging)
Data analysis	SoftWoRx 3.0 (Applied Precision); ZEN Black 2012 SP4, version 13.0.2.518 (Carl Zeiss Microimaging);<br>Adobe photoshop CC version 20; Fiji version 2.0.0-rc-59/1/51k; Imaris version 9.5.1; excel 16.16, Graphpad Prism 8.<br>bcl2fastq (version 2.1.7); using Tophat2 version 2.0.11 ; featureCounts from Rsubread package version 1.20.6

For manuscripts utilizing custom algorithms or software that are central to the research but not yet described in published literature, software must be made available to editors and reviewers. We strongly encourage code deposition in a community repository (e.g. GitHub). See the Nature Research guidelines for submitting code & software for further information.

Data

Policy information about availability of data

All manuscripts must include a data availability statement. This statement should provide the following information, where applicable:

- Accession codes, unique identifiers, or web links for publicly available datasets
- A list of figures that have associated raw data
- A description of any restrictions on data availability

RNAseq data set for Fig 5A can be accessed using GEO accession GSE134989 (https://www.ncbi.nlm.nih.gov/geo/query/acc.cgi?acc=GSE134989). The source data underlaying Figs 3A-E, Figs 4B, D, E, Figs 5A, B, C, D, F, G, H, Fig 6B, Fig 7B, Supplementary Figs 2C, D, J, Supplementary Figs 3B-E, Supplementary Figs 4B-E, and Supplementary Figs 5D, E, G are provided as a Source Data file. All other relevant data are available within the Article and Supplementary Information files or available from the authors upon reasonable request. C. elegans strains generated in this study are available from the corresponding author.

Field-specific reporting

Please select the one below that is the best fit for your research. If you are not sure, read the appropriate sections before making your selection.

☒ Life sciences ☐ Behavioural & social sciences ☐ Ecological, evolutionary & environmental sciences

For a reference copy of the document with all sections, see nature.com/documents/nr-reporting-summary-flat.pdf

Life sciences study design

All studies must disclose on these points even when the disclosure is negative.

Sample size	No statistical methods were used to predetermine sample size. Sample sizes were determined based on estimates from preliminary data and on similar studies where whole C elegans germlines were imaged and used for quantifications. We quantified 3 germlines per genotype/condition with each collected germline containing around 300 nuclei between leptotene and late pachytene, therefore each bar plot includes data from around 900 nuclei per condition/genotype.
Data exclusions	No data was excluded for analysis
Replication	All data were suitably replicable. All microscopy experiments were performed at least as two independent replicates per condition/genotype. Between 5 to 20 germlines were observed in each replicate per condition/genotype.
Randomization	For TEV and auxin treatment experiments individual worms were picked from plates containing large populations and randomly divided into controls and worms used for TEV/auxin treatment.
Blinding	Experiments were performed in a non-blinded manner, the same person that performed condition/genotype variations also imaged dissected germlines.

Reporting for specific materials, systems and methods

We require information from authors about some types of materials, experimental systems and methods used in many studies. Here, indicate whether each material, system or method listed is relevant to your study. If you are not sure if a list item applies to your research, read the appropriate section before selecting a response.

Materials & experimental systems

n/a	Involved in the study	
☐	☒	Antibodies
☒	☐	Eukaryotic cell lines
☒	☐	Palaeontology and archaeology
☐	☒	Animals and other organisms
☒	☐	Human research participants
☒	☐	Clinical data
☒	☐	Dual use research of concern

Methods

n/a	Involved in the study	
☒	☐	ChIP-seq
☒	☐	Flow cytometry
☒	☐	MRI-based neuroimaging

Antibodies

Antibodies used	rabbit anti-GFP-488-conjugated (Invitrogen, A21311); goat anti-GFP-FITC-conjugated (Abcam, ab6662); rat anti-mCherry (Chromotek, 5F8); mouse anti-REC-8 (Novus Biologicals, 29470002); rabbit anti-COH-3/4 (Crawley et al. (2016). eLife 5, e10851); chicken anti-SYP-1 (Silva et al. (2014) Dev Cell 31, 503-511); guinea pig anti-SYP-1 (MacQueen et al. (2002) Genes Dev 16, 2428-2442); rabbit anti-HTP-1/2 (Silva et al. (2014) Dev Cell 31, 503-511); guinea pig anti-HTP-3 (Goodyer et al. (2008) Dev Cell 14, 263-274); rabbit anti-HIM-3 (Zetka et al. (1999) Genes Dev 13, 2258-2270); rabbit anti-PLK-2 (Nishi et al. (2008) Development 135, 687-697); guinea pig anti-SUN-1 pS12 (Woglar et al. (2013) PLoS Genet 9, e1003335); rabbit anti-DSB-2 (Rosu et al. (2013) PLoS Genet 9, e1003674); rabbit anti-RAD-51 (Novus Biologicals, 29480002), mouse anti-HA (Cell Signalling, 23675); rabbit anti-HIM-8 (Novus Biologicals, 41980002); goat anti-actin (Santa Cruz, SC1616); HRP-conjugated donkey anti-goat IgG (Sigma, AP180P); HRP-conjugated goat anti-rabbit (Jackson Immunoresearch, AB_2307391).

Validation	Phospho-specific antibodies generated in this study against HIM-8 pT64 were validated by lack of staining in animals lacking CHK-2 activity.<br>Validation statements of commercial available antibodies are on the manufacturer's website:<br>goat anti-actin (Santa Cruz, SC1616) https://www.scbt.com/p/actin-antibody-i-19<br>rabbit anti-HIM-8 (Novus Biologicals, 41980002) https://www.novusbio.com/products/him-8-antibody_41980002<br>rabbit anti-GFP-488-conjugated (Invitrogen, A21311). https://www.thermofisher.com/antibody/product/GFP-Antibody-Polyclonal/A-21311<br>rat anti-mCherry (1:1000) (Chromotek, 5F8) https://www.chromotek.com/products/detail/product-detail/rfp-antibody-5f8/<br>goat anti-GFP-FITC-conjugated https://www.abcam.com/gfp-antibody-fitc-ab6662.html

Animals and other organisms

Policy information about studies involving animals; ARRIVE guidelines recommended for reporting animal research

Laboratory animals	C. elegans strains were used on this study (Table S3). Worms were dissected at 18-24 hours post L4.
Wild animals	No wild animals were used in this study
Field-collected samples	No field collected samples were used in this study
Ethics oversight	No ethical approval required for C. elegans work

Note that full information on the approval of the study protocol must also be provided in the manuscript.
